# Epigenetic regulation of spinal cord gene expression controls Opioid-Induced Hyperalgesia

**DOI:** 10.1186/1744-8069-10-59

**Published:** 2014-09-12

**Authors:** De-Yong Liang, Yuan Sun, Xiao-You Shi, Peyman Sahbaie, J David Clark

**Affiliations:** Department of Anesthesia, Stanford University School of Medicine, Stanford, CA 94305 USA; Anesthesiology Service, Veterans Affairs Palo Alto Health Care System, 3801 Miranda Ave (112-A), Palo Alto, CA 94304 USA

**Keywords:** Epigenetics, Histone acetylation, Opioid induced hyperalgesia, BDNF and dynorphin

## Abstract

**Background:**

The long term use of opioids for the treatment of pain leads to a group of maladaptations which includes opioid-induced hyperalgesia (OIH). OIH typically resolves within few days after cessation of morphine treatment in mice but is prolonged for weeks if histone deacetylase (HDAC) activity is inhibited during opioid treatment. The present work seeks to identify gene targets supporting the epigenetic effects responsible for OIH prolongation.

**Results:**

Mice were treated with morphine according to an ascending dose protocol. Some mice also received the selective HDAC inhibitor suberoylanilide hydroxamic acid (SAHA) additionally. Chronic morphine treatment with simultaneous HDAC inhibition enhanced OIH, and several spinal cord genes were up-regulated. The expression of *Bdnf* (Brain-derived neurotrophic factor) and *Pdyn* (Prodynorphin) were most closely related to the observed behavioral changes. ChIP (Chromatin immuoprecipation) assays demonstrated that promoter regions of *Pdyn* and *Bdnf* were strongly associated with aceH3K9 (Acetylated histone H3 Lysine9) after morphine and SAHA treatment. Furthermore, morphine treatment caused an increase in spinal BDNF and dynorphin levels, and these levels were further increased in SAHA treated mice. The selective TrkB (tropomyosin-receptor-kinase) antagonist ANA-12 reduced OIH when given one or seven days after cessation of morphine. Treatment with the selective kappa opioid receptor antagonist nor-BNI also reduced established OIH. The co-administration of either receptor antagonist agent daily with morphine resulted in attenuation of hyperalgesia present one day after cessation of treatment. Additionally, repeated morphine exposure induced a rise in BDNF expression that was associated with an increased number of BDNF^+^ cells in the spinal cord dorsal horn, showing strong co-localization with aceH3K9 in neuronal cells. Lastly, spinal application of low dose BDNF or Dynorphin A after resolution of OIH produced mechanical hypersensitivity, with no effect in controls.

**Conclusions:**

The present study identified two genes whose expression is regulated by epigenetic mechanisms during morphine exposure. Treatments aimed at preventing the acetylation of histones or blocking BDNF and dynorphin signaling may reduce OIH and improve long-term pain using opioids.

## Background

Opioids have long been accepted as a critical treatment option for acute forms of pain. As useful as these medications may be in acute settings, however, a specific group of maladaptations limits the long term effectiveness of these drugs including the phenomena of analgesic tolerance, physical dependence and opioid-induced hyperalgesia (OIH) [1–3]. Moreover, there has been a very sharp rise in the rate of opioid abuse paralleling the rise in their prescription for the treatment of pain, and opioid abuse has been recognized by the CDC as a major public health epidemic in the US [4]. Much recent effort has been placed on the study of OIH as patients exposed to opioids through illicit use or for legitimate medical indications for sustained periods demonstrate heightened pain sensitivity to experimental pain stimuli, pain during minor procedures and pain after major surgery [5–9]. Many genes contribute the regulation of opioid adaptations and OIH [10–12], though mechanisms coordinating the expression of these genes are very poorly described. It is possible that by understanding overarching regulatory mechanisms, we will be in better position to prevent opioid maladaptations or mitigate their impact on our long term pain control goals.

Epigenetics refers to a collection of processes controlling how the genome is used without altering DNA sequence [13–16]. Many of these processes control long term and sometimes heritable changes in gene expression. Some of the more commonly studied mechanisms include the control of covalent histone modifications (acetylation, methylation and etc.), DNA methylation and miRNA synthesis. One key feature of these epigenetic mechanisms is that they mediate the responses of specific cell and tissue types within an organism to changes in environmental factors. These factors including nutrients, drugs, stress and others control the expression of groups of genes simultaneously rather than single genes specifically. Examining the influence of these environmental factors on concurrent control over expression of several genes has moved the study of epigenetics to the forefront of addiction and pain-related research. For example, the study of cocaine effects on changes in gene expression in the addiction-related nucleus accumbens showed that this drug changed the expression of *Fosb*, *Camk2a*, *Cdk5*, *Bdnf* and other addiction-related genes via alterations in histone acetylation [17]. A recent set of studies from our laboratory showed that alterations in morphine-induced histone acetylation in spinal cord tissue helped to regulate morphine tolerance, dependence and OIH [18], though gene targets for these epigenetic effects were not characterized.

Here, we present studies addressing the hypothesis that morphine induces OIH via the regulation of histone acetylation controlling the expression of specific genes in spinal cord tissue. We selected for these studies a well-characterized mouse model of tolerance, dependence and OIH used previously to discover genetic and biochemical factors controlling these maladaptations [18–22]. We demonstrated in the same mouse model that OIH largely resolves within 7 days after cessation of morphine administration, but inhibition of histone deacetylase (HDAC) activity during morphine treatment prolonged sensitization for weeks. The present work will follow up on our previous report to further characterize the genes responsible for the observed sensitization. Also, it seems reasonable to focus on the identification of genes regulated by epigenetic mechanisms during morphine exposure as an approach to understanding mechanisms underpinning OIH.

## Results

### Epigenetic effects of chronic morphine treatment on spinal cord gene expression

**Figure 1:**
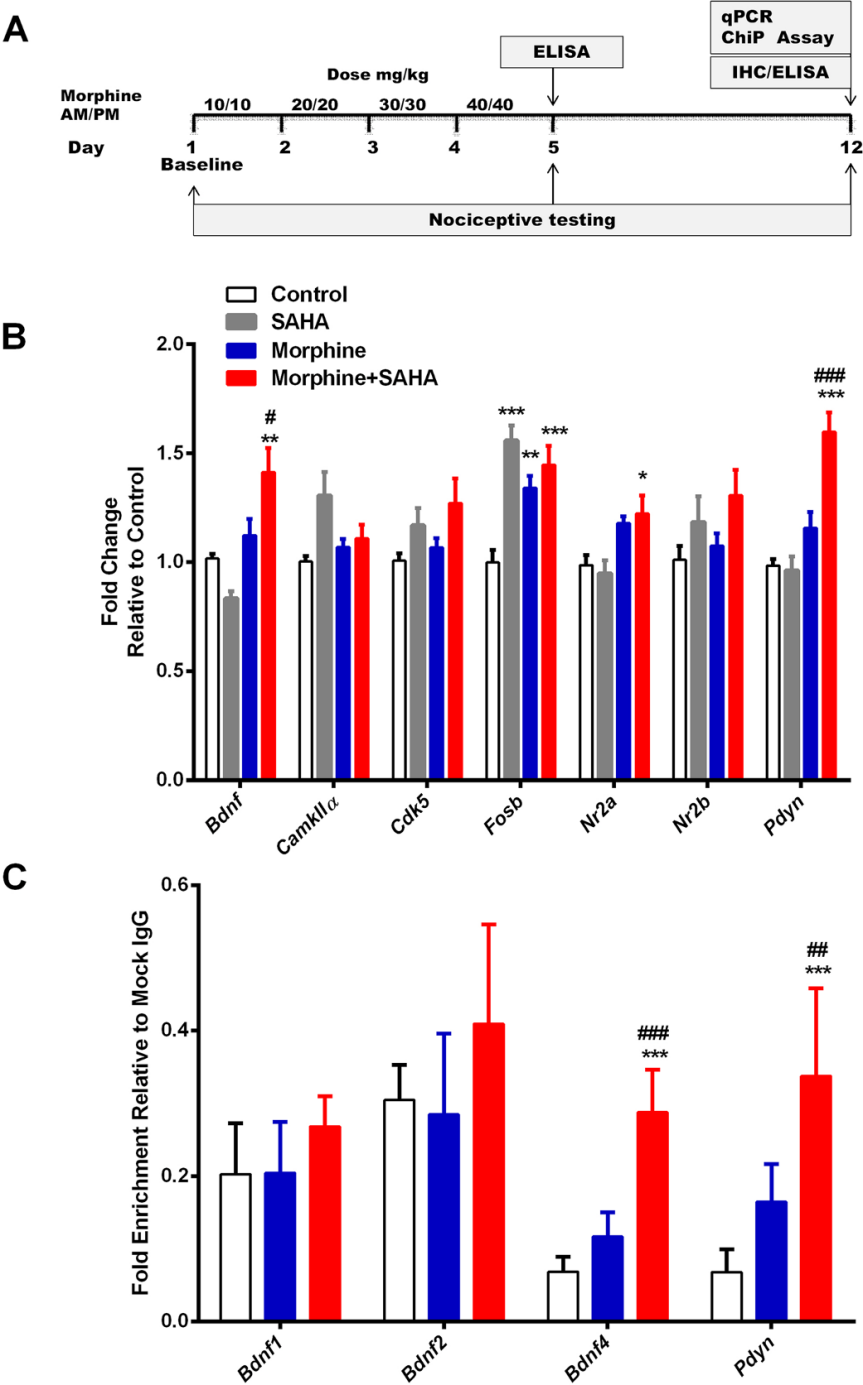
**Epigenetic effects of chronic morphine treatment on spinal cord gene expression. (A)** Schematic representation of experimental timeline showing the daily dosing schedule of morphine treatment. **(B-C)** Epigenetic effects of escalating dose morphine treatment on spinal cord gene expression changes after 7 days of washout with/without co-administration of SAHA 50 mg/kg daily. Concomitant HDAC inhibition leads to significant up-regulation in *Bdnf* and *Pdyn* mRNA expression 7 days after cessation of morphine treatment **(B)**. The promoter regions of *Pdyn* and *Bdnf* exon-IV genes were more strongly associated with acetylated histone H3: aceH3K9 **(C)**. Error bars: SEM, n = 6-8/group, ^*^p < 0.05, **p < 0.01 and ***p < 0.001 for comparison with controls. ^#^p < 0.05, ^##^p < 0.01 and ^###^p < 0.001 for comparison with morphine group. Data were analyzed by two-way ANOVA followed by Bonferroni post-hoc tests.

The experimental timeline showing the daily dosing schedule of morphine treatment is represented schematically in Figure 1A. Here we investigated the effects of morphine treatment on the expression of a panel of genes implicated previously in maladaptations to opioids: *Fosb*, *Camk2a*, *Cdk5*, *Bdnf, Pdyn* and *Grin2a* in spinal cord tissue 7 days after cessation of morphine treatment with and without co-administration of SAHA [17, 18, 23]. We found significant and sustained up-regulation of several genes. However, it was only for *Bdnf* and *Pdyn* that levels were higher after simultaneous SAHA and morphine treatment than morphine or SAHA treatment alone indicating a strong epigenetic effect (Figure 1B). The *Bdnf* levels following SAHA or morphine treatments were not different from controls (*p* = 0.36 and *p* = 0.71 vs. controls respectively, or each other *p* = 0.10). Similarly, *Pdyn* levels following SAHA or morphine treatments were not different from controls (*p* = 0.99 and *p* = 0.21 vs. controls respectively, or each other *p* = 0.21). As SAHA alone treatment failed to sustain a significant up-regulation of either gene, subsequent analyses were conducted comparing morphine and morphine plus SAHA groups. Moreover, ChIP analysis demonstrated that the promoter regions of *Pdyn* and *Bdnf* (Exon 4 variant) were more strongly associated with aceH3K9 (Acetylated histone H3 Lysine9) after morphine and SAHA treatment than when animals were treated with morphine alone (Figure 1C). There were no significant differences in levels of enrichment for *Pdyn* (*p* = 0.11) or *Bdnf* Exon 4 (*p* = 0.13) between vehicle and morphine treatments. The rodent Bdnf gene contains 9 upstream exons which can give rise to various splice variants, with Bdnf mRNA s containing exon 1,2 and 4 being more transcriptionally important [24, 25]. Although the explicit function of each transcript is unknown, region specific differential expression of exons 1 and 4 variants in the nervous system has been observed after cocaine abstinence [26, 27]. The present study implicates exon 4 variant to be differentially expressed in spinal cord following epigenetically prolonged OIH.

### Effects of HDAC inhibitor treatment on spinal cord BDNF and dynorphin protein levels after morphine treatment

**Figure 2:**
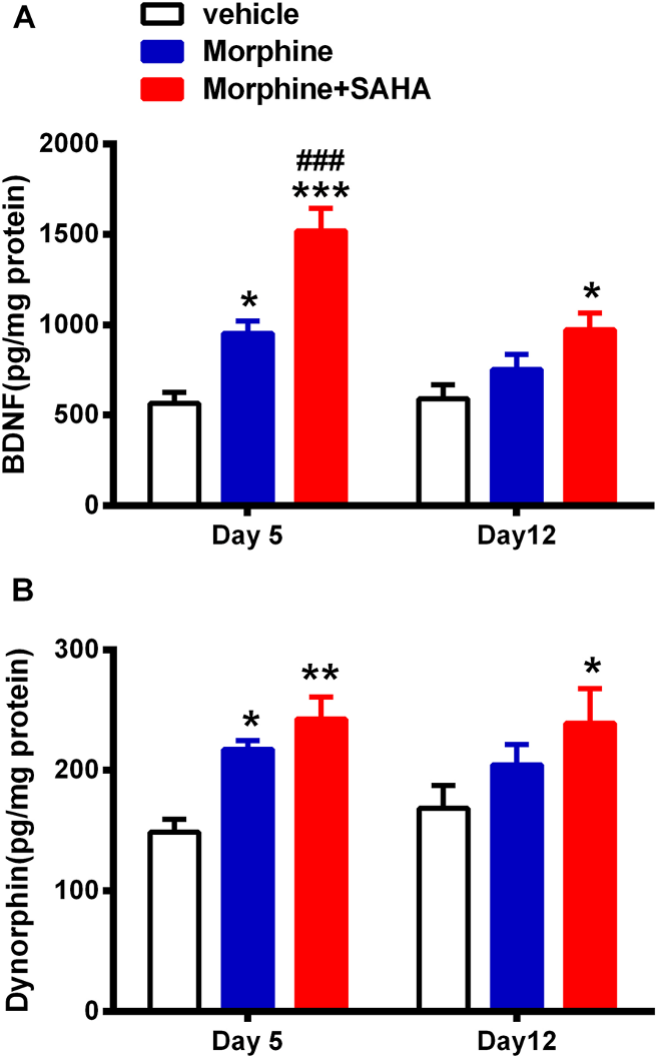
**HDAC inhibitor treatment increases spinal cord BDNF and dynorphin protein levels after morphine treatment.** SAHA treatment resulted in significantly increased expression of BDNF after 1 or 7 days since cessation of morphine treatment compared to vehicle treatment **(A)**. Levels of dynorphin were similarly increased on day 5 in SAHA and vehicle groups and it was significantly up-regulated 7 days after morphine and SAHA treatment only **(B)**. HDAC inhibition was carried out for the duration of escalating dose morphine administration. Error bars: SEM, n = 5-6/group; *p < 0.05, **p < 0.01, ***p < 0.001 for comparison with vehicle and ^###^p < 0.001 for comparison with morphine treatment. Data were analyzed by two-way analysis of variance (ANOVA) followed by Tukey’s post-hoc test for multiple comparisons within each timepoint.

Next we determined whether the effects of HDAC inhibition during morphine administration on *Bdnf* and *Pdyn* gene expression translated to sustained increases in mediator protein levels in spinal cord tissue. Figure 2A shows morphine treatment alone caused an increase in the spinal level of BDNF observable 1 day (Day 5) after the cessation of morphine treatment, but that the spinal cord BDNF level was similar to control by 7 days (Day 12). The inclusion of SAHA resulted in significantly increased expression of BDNF at both time points. The time course of morphine-enhanced spinal cord dynorphin levels was similar to that of BDNF, and the inclusion of SAHA with morphine again prolonged the enhanced dynorphin levels (Figure 2B). Together with the data in Figure 1, these observations suggest that the acetylation of H3K9 near the promoter regions of the *Bdnf* and *Pdyn* genes controls their expression during and after chronic morphine administration.

### Selective inhibition of BDNF and dynorphin signaling attenuates opioid induced hyperalgesia

**Figure 3:**
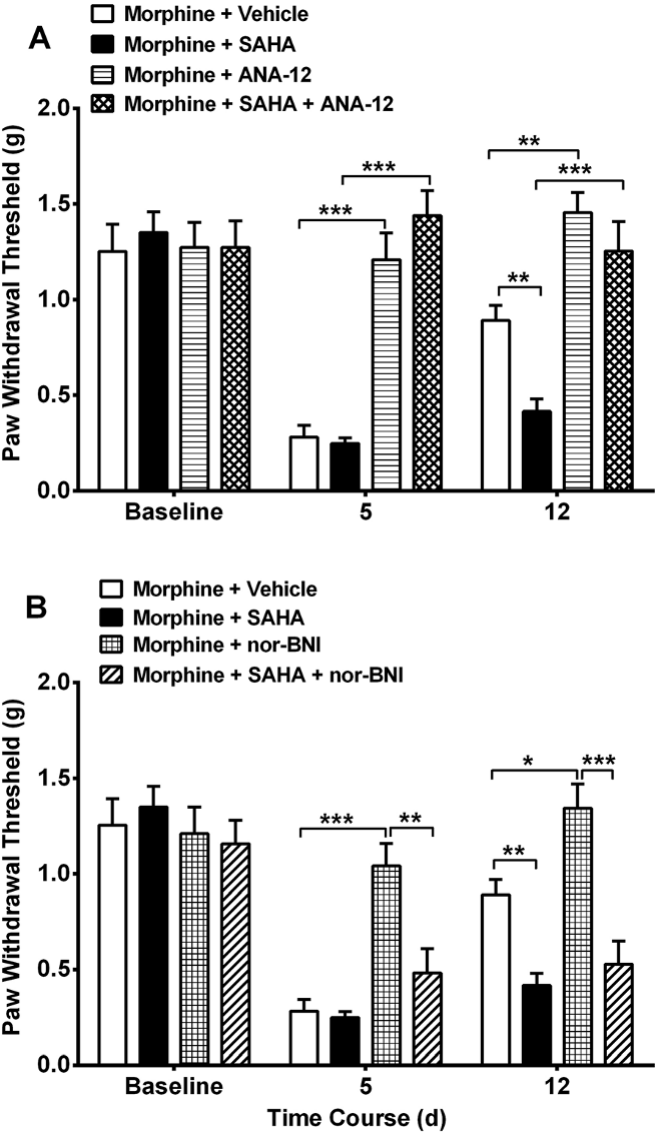
**Selective acute inhibition of BDNF and prodynorphin signaling attenuates opioid induced hyperalgesia.** Effects of single dose ANA-12 (TrkB receptor antagonist, 1 mg/kg) or nor-BNI (KOR antagonist, 1 mg/kg) in the setting of OIH was examined. Selective antagonism of TrkB **(A)** receptor and KOR **(B)** produces a reversal of mechanical hypersensitivity, whether given once at day 5 (day 1 after completion of morphine protocol) or day 12 (day 7 after completion of morphine). However, acute ANA-12 but not nor-BNI administration reversed the sensitization present in the morphine + SAHA group. Error bars: SEM, n = 6/group, *p < 0.05, **p < 0.01 and ***p < 0.001 for comparison between treatments for each timepoint. Data for each timepoint were analyzed by one-way ANOVA followed by Sidak multiple comparisons tests.

In order to test the hypothesized functional links between BDNF, dynorphin and OIH, we used pharmacological tools. Specifically, we used selective antagonists of the TrkB (tropomyosin-receptor-kinase) and κ-opioid receptor (KOR), ANA-12 and nor-BNI respectively. Figure 3A-B show that selective antagonism of the TrkB receptor reduced mechanical sensitization given on day 1 or 7 after cessation of morphine (*p* < 0.001) or morphine + SAHA (*p* < 0.001) compared to vehicle treatment. No significant differences were observed for ANA-12 treatment between morphine or morphine + SAHA groups (*p* = 0.54). Treatment with nor-BNI was not sufficient to reverse hypersensitivity given as a single dose to mice previously treated with morphine plus SAHA (*p* = 0.59), though it could reverse sensitization in mice treated with morphine alone (*p* < 0.001) compared to vehicle treatment.

**Figure 4:**
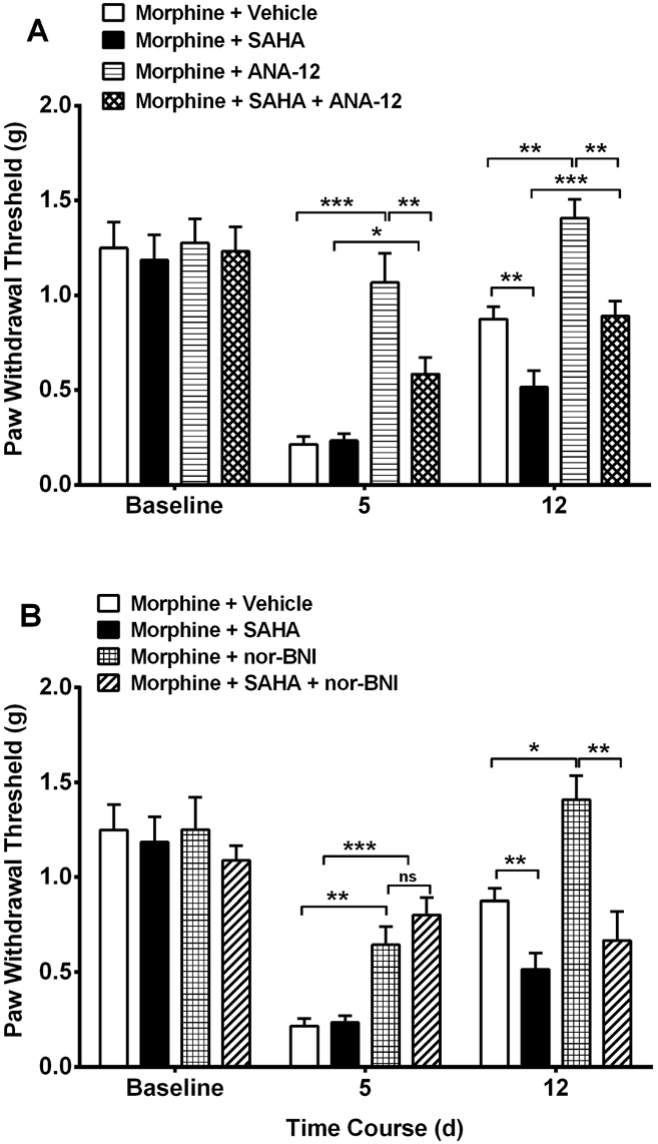
**Selective inhibition of BDNF and prodynorphin signaling during morphine treatment attenuates opioid induced hyperalgesia.** ANA-12 (TrkB receptor antagonist, 0.5 mg/kg) or nor-BNI (KOR antagonist, 0.5 mg/kg) administration once daily with morphine during the 4 days of escalating morphine treatment resulted in attenuation of sensitization on day 5 **(A-B)**. However, only ANA-12 was associated with less sensitization in the morphine + SAHA group on day 12 (one week after completion of drug administration). Error bars: SEM, n = 6/group, ns: p > 0.05, *p < 0.05, **p < 0.01 and ***p < 0.001 for comparison between treatments for each timepoint. Data for each timepoint were analyzed by one-way ANOVA followed by Sidak multiple comparisons tests.

The co-administration of either receptor antagonist agents daily with morphine or morphine plus SAHA for 4 days resulted in attenuation of hyperalgesia on day 5. Seven days after cessation of drug treatment ANA-12 treated animals that had received morphine (*p* < 0.001) or morphine plus SAHA (*p* < 0.001) continued to have less mechanical hypersensitivity compared to vehicle treatment. Though, significant differences were observed for ANA-12 treatment between morphine and morphine + SAHA groups (*p* < 0.01), the latter group showed significant attenuation of hyperalgesia compared to vehicle treatment (*p* < 0.01, Figure 4A). However, only nor-BNI treated animals that received morphine continued to have less mechanical hypersensitivity (*p* < 0.05 vs. vehicle group). The nor-BNI treatment in morphine plus SAHA treatment group did not produce a lasting effect on hypersensitivity (*p* = 0.68, Figure 4A).

### Increased spinal cord histone acetylation levels after morphine treatment promotes BDNF expression

**Figure 5:**
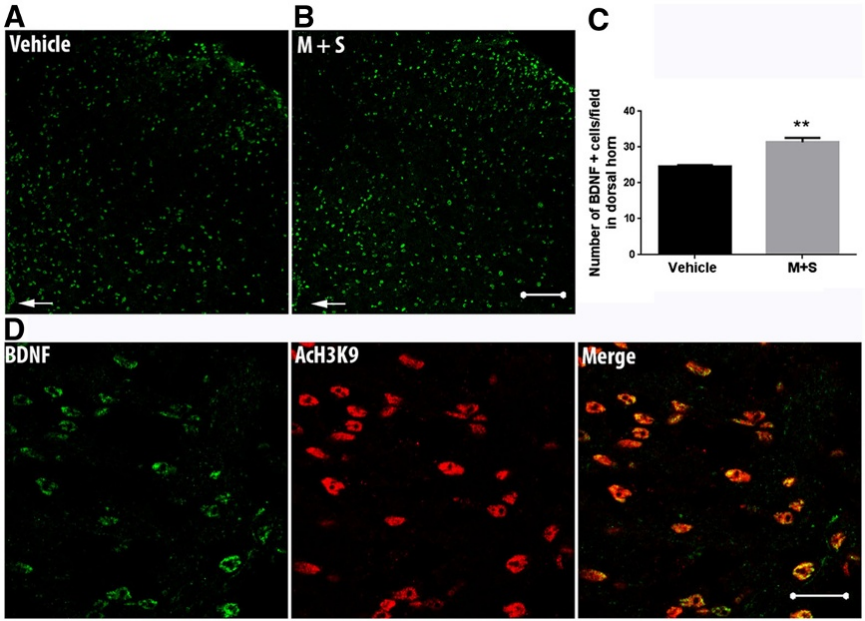
**Chronic morphine with SAHA treatment promotes histone acetylation dependent increase in BDNF expression in spinal cord tissue. (A-B)** Micrographs of representative spinal cord dorsal horn sections (Scale bar = 100 micron) showing increased number of BDNF^+^ cells in the dorsal horn 7 days after cessation of morphine and SAHA (M + S) treatment. **(C)** Quantification of BDNF positive cells in 10–15 randomly (2 slices per mouse) selected high-power fields (HPF, 400X) of spinal cord dorsal horn per animal. **(D)** BDNF and acetylated H3K9 co-staining in the M + S treatment group, demonstrating strong association between both. Error bars: SEM, n = 4/treatment group. Data analyzed by unpaired two tailed student *t*-tests, **p < 0.01 for differences between treatment conditions.

**Figure 6:**
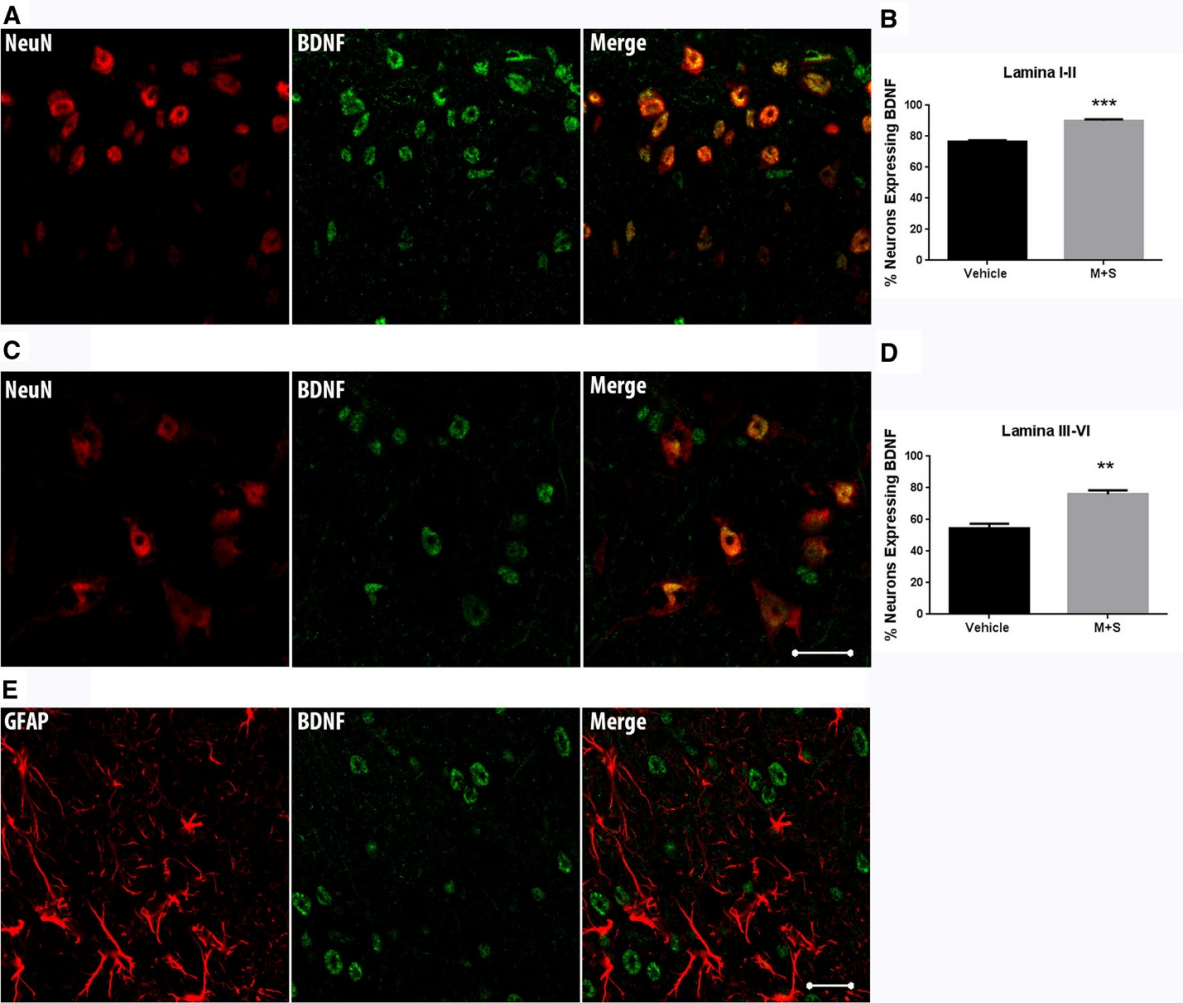
**Chronic morphine with SAHA treatment increases the number of BDNF** ^**+**^ **expressing spinal cord dorsal horn neurons. (A-C)** Double staining for BDNF and NeuN markers demonstrates strong association with BDNF in neuronal cells (Scale bar = 20 micron). **(B-D)** Quantification of double staining for BDNF and NeuN markers reveals increased percentage of neurons expressing BDNF in lumbar spinal cord dorsal horn tissue (lamina I-II and III-VI) following M + S treatment. **(E)** Double staining for BDNF and GFAP markers shows less abundant BDNF expression by astrocytes. Error bars: SEM, n = 4/ treatment group with the numbers of NeuN positive cells (neurons) and BDNF positive neurons were counted in 10–15 randomly selected (2 slices per mouse) high-power fields (HPF, 400X) of spinal cord dorsal horn per animal. Data analyzed by unpaired two tailed student *t*-tests, **p < 0.01 and ***p < 0.001 for differences between treatment conditions.

Because our expression data suggested spinal BDNF expression may be particularly important for OIH after chronic opioid administration, we determined whether this mediator is specifically associated with aceH3K9 containing cells in the spinal cord dorsal horn. Figure 5A-C show an increased number of BDNF^+^ cells in the dorsal horn quantified 7 days following completion of morphine plus SAHA treatment. Additionally, Figure 5D demonstrates strong co-localization BDNF and aceH3K9 using double-labeling techniques. To further characterize the cell types responsible for the increased BDNF production, double staining techniques using NeuN (neurons) and GFAP (astrocytes) markers. Figures 6A-D show increased neuronal production of BDNF after morphine and SAHA treatment. Additionally, the observed increase was not strongly associated with spinal astrocytes (Figure 6E). Further quantification revealed 82.14 ± 2.05 percent of neuronal cells and 6.08 ± 0.44 percent of astrocytes in lamina I-II to have BDNF positive markers 7 days after completion of morphine and SAHA treatment. For lamina III-VI, 59.68 ± 3.49 percent of neuronal cells and 5.35 ± 0.61 percent of astrocytes in lamina I-II were BDNF positive for the same time point.

### Intrathecal low dose BDNF and dynorphin A produce hyperalgesia in resolved OIH

**Figure 7:**
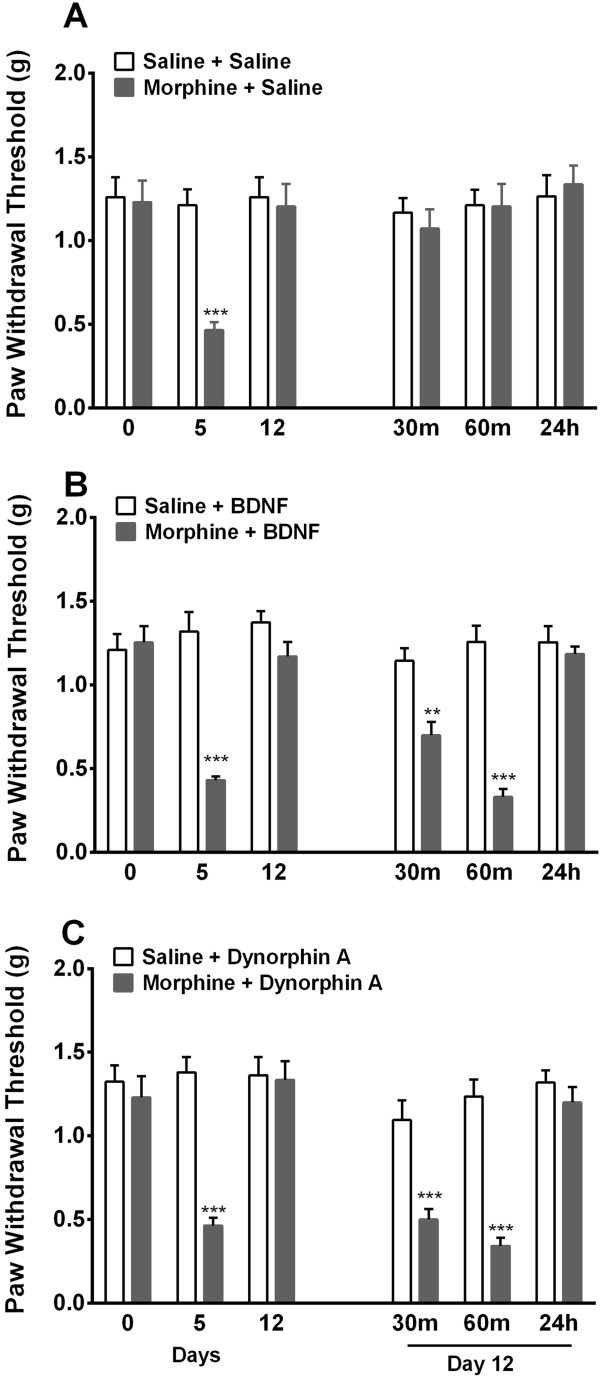
**Spinal application of low dose BDNF or dynorphin A produces hyperalgesia in previously morphine treated mice.** Effects of single intrathecal dose of **(A)** saline (5uL), **(B)** BDNF 1 ng/5uL or **(C)** Dynorphin A 1 nmol (5uL) in mice 7 days after cessation of morphine/saline treatment OIH were examined. The drug doses selected fail to produce mechanical hypersensitivity in animals previously treated with saline. However, in the setting of resolved OIH both agents produce robust mechanical allodynia. Error bars: SEM, n = 6/group, ^**^p < 0.01 and ^***^p < 0.001 for comparison between treatments for each timepoint. Data for each timepoint were analyzed by two-way ANOVA followed by Sidak multiple comparisons tests.

Here we investigated the effects of single intrathecal dose of BDNF or dynorphin A in mice 7 days after cessation of morphine treatment. The experiments were carried out to determine if the increased protein levels of BNF or dynorphin were directly responsible for prolonged OIH after morphine and SAHA treatments. Figure 7A shows spinal vehicle treatment to have no effect on resolved OIH. However application of BDNF 1 ng/5mcl or Dynorphin A 1 nmol (5mcl) in mice 7 days after cessation of morphine treatment produced robust mechanical hypersensitivity (Figure 7B-C). The drug doses selected failed to produce mechanical hypersensitivity in animals previously treated with saline. The same dose of either agent administered intrathecally has been shown to produce no significant hyperalgesia in naïve animals [28, 29].

## Discussion

Our growing understanding of epigenetic mechanisms has motivated investigations of the roles of these processes in many scientific fields relevant to anesthesiology and pain management including those involving pain mechanisms and addiction. Our studies were intended to build on those efforts by refining our understanding of how histone acetylation, a particularly well established type of epigenetic process, might alter opioid-induced hyperalgesia (OIH), a problematic consequence of chronic opioid administration [10–12]. We began our studies by selecting a panel of established genes known to mediate the consequences of chronic opioid administration such as OIH, tolerance, physical dependence and addiction in CNS tissues. The selected genes were analyzed further to find candidates most likely to be under epigenetic control in spinal cord tissue. The spinal cord is a critical site of action for opioids with respect to analgesia, and is perhaps the best studied site of action for mechanisms related to OIH [10]. Those analyses suggested that both the *Bdnf* and *Pdyn* genes might undergo morphine-induced changes in expression attributable to histone acetylation. Further pursuit of these genes revealed that selective antagonists for BDNF (tropomyosin receptor kinase-B, TrkB) or dynorphin (κ-opioid receptor, KOR) receptors could in fact block OIH. Further immunohistochemical studies localized the spinal expression of BDNF mostly to neurons within the dorsal horn regions of the spinal cords of mice. Lastly, spinal application of low dose BDNF or dynorphin produced heperalgesia when OIH was resolved, while having no effect in opioid naïve groups.

In these studies the TrkB receptor ligand BDNF was strongly linked to OIH using gene expression, chromatin immunoprecipitation, immunohistochemical and pharmacological data. This neurotrophin has been studied in association with learning, memory and drug addiction. Others have suggested that BDNF expressed in various nervous system tissues exerts actions either facilitating or countering those of morphine and other opioids. For example, Meng et al. recently used the conditioned place preference model of opioid addiction to determine that BDNF expression in the hippocampus, caudate/putamen and nucleus accumbens supports drug seeking behavior in mice [30]. Likewise the administration of low doses of morphine to neonatal rats caused changes in hippocampal BDNF expression into adult life suggesting the involvement of long term epigenetic adaptive mechanisms [31]. Consistent with the notion that *Bdnf* expression might be controlled by histone acetylation, the regulation of *Bdnf* expression through histone acetylation in prefrontal cortex was observed to mediate the negative affective component of morphine withdrawal memory in rats [32]. Similarly, cocaine exposure enhanced *Bdnf* expression in the nucleus accumbens through enhanced histone acetylation as well [17]. Our own recent studies and those of others demonstrated the up-regulation of *Bdnf* in spinal cord tissue after several days of morphine exposure in rats and mice [18, 33, 34]. The studies of Ferrini et al. demonstrated a strong role for BDNF in OIH. In these studies BDNF released from spinal dorsal horn microglia was shown to down-regulate expression of the KCC2 K^+^-Cl^-^ co-transporter in lamina I projection neurons [33]. The immunohistochemical studies done here showed that the majority of the BDNF in our animals was neuronal in origin, though we cannot exclude a functionally relevant amount of BDNF in glia which has been suggested by others to participate in opioid tolerance and dependence [33, 34]. Although transcription of Bdnf is controlled by multiple promoters, activity dependent increase in exon 1 and 4 containing transcripts in the brain contributes significantly to neuronal plasticity [35]. Also, another study has shown BDNF exon 1 containing transcripts to be up-regulated in the ventral tegmental area following cocaine abstinence [27]. Our study shows the exon 4 variant to be differentially expressed in spinal cord following epigenetically prolonged OIH. Furthermore, while we did not study second messenger system activation downstream of BDNF production, we did demonstrate that the TrkB antagonist ANA-12 was very effective in reversing morphine-related OIH. Similarly, identification of spinal second order TrkB expressing projection neurons mediating such second messenger activation after chronic morphine treatment though not investigated here, would be of interest [36, 37].

The second gene from the spinal multi-gene panel found to be associated with morphine-induced epigenetic regulation was *Pdyn* coding for prodynorphin. The translation of this gene and subsequent processing ultimately leads to the production of dynorphin A, dynorphin B and other peptides. Dynorphin A has a particularly complex pharmacological profile in that it interacts with both opioid and non-opioid receptors in spinal pain signaling pathways [38, 39]. Though prodynorphin products can be released from afferent sensory nerve terminals, the *Pdyn* gene is strongly expressed by spinal cord dorsal horn GABAergic interneurons [40], and these are a possible source of the *Pdyn* mRNA measured in our experiments. Immunohistochemical studies of dynorphin expression in our animals did not reveal a clear cellular source (data not shown). Spinal prodynorphin and its component peptides have been identified as supporting pain in several types of models including chronic neuropathic [41, 42] and inflammatory pain [43, 44]. It is also the case that epigenetic regulation of the *Pdyn* gene has been demonstrated [45]. Most pertinent to present work, spinal *Pdyn* expression and dynorphin abundance have been functionally linked to OIH in rodent studies, though the mechanisms responsible for its up-regulation are not as clearly described [41, 46–48]. We demonstrated that morphine-induced up-regulation of prodynorphin was epigenetically mediated as supported by ChIP experiments and results obtained when using a HDAC inhibitor to pharmacologically increase histone acetylation. The use of the selective KOR antagonist did reduce OIH when given acutely after cessation of morphine administration or when given along with daily doses of morphine. On the other hand, the selective KOR antagonist was less effective when animals had been treated with both morphine and the HDAC inhibitor SAHA. This may be attributable to the possibility that higher levels of dynorphin peptides present when morphine and SAHA were administered were acting through other receptors such as the B2 bradykinin receptor to enhance nociception [38]. Thus while spinal KOR signaling may be involved, peptides derived from epigenetically regulated *Pdyn* expression may also participate in supporting OIH via additional non-opioid receptor mechanisms.

The present studies identified *Bdnf* and *Pdyn* as genes epigenetically regulated through the acetylation of histone proteins consistent with other’s observations. Uchida et al., for example, reported that histone acetylation affected *Bdnf* transcription after peripheral nerve injury [49]. Both histone proteins H3 and H4 seemed to be involved in these nerve injury-induced changes whereas only H3 was observed to undergo morphine-induced acetylation in our model [18]. Though this regulation was demonstrated after nerve injury to take place in peripheral nerves rather than spinal cord tissue, the net effect of the up-regulation was to enhance BDNF levels in the spinal cord, and to support nociceptive sensitization. We did not examine sensory neurons for changes in *BDNF* expression after morphine exposure in our experiments. Interestingly, chronic ethanol exposure leads to the development of neuroplasticity in the amygdala and the up-regulation of *Pdyn* expression possibly by enhanced acetylation of *Pdyn* promoter H3 protein at the K9 position similar to what we observed for the morphine-related effects [45]. Thus similar epigenetic mechanisms may control *Bdnf* and *Pdyn* expression in different neural tissues to alter nociception, analgesic sensitivity and drug-induced changes in emotions.

## Conclusions

The present studies have identified two specific spinal cord genes whose expression is regulated by epigenetic mechanisms occurring during morphine exposure. Though it is unlikely that these are the only genes epigenetically regulated during chronic morphine treatment, both *Bdnf* and *Pdyn* possibly play a roles in OIH and other maladaptations to opioids. It may be reasonable, then, to consider treatments preventing the acetylation of histones as viable ones in preventing OIH when long term opioid therapy is required. On the other hand, we do not at this point know whether agents can be developed that reduce only epigenetically-regulated opioid maladaptations and leave intact the many other cellular processes that might to some degree be epigenetically regulated as well. More broadly, epigenetic processes such as histone acetylation may be responsible for integrating the effects of environmental factors like drug administration with intrinsic processes like pain signaling pathways to cause long lasting alterations in neuronal function. Models similar to those used here could be used to explore this possibility.

## Methods

### Animal subjects

All animal experiment protocols were approved by the Veterans Affairs Palo Alto Health Care System Institutional Animal Care and Use Committee (Palo Alto, California, United States) and complied with the Guide for the Care and Use of Laboratory Animals available through the National Academy of Sciences. Male C57BL/6J mice were obtained from Jackson Laboratory (JAX, Bar Harbor, ME) at 7–8 weeks of age. Experiments were done after a 7–10 day acclimation period in our animal care facility. Mice were housed 4 per cage under pathogen-free conditions with soft bedding and were provided food and water ad libitum with a 12:12 light: dark cycle.

### Chronic morphine administration

After baseline nociceptive testing, morphine (Sigma Chemical, St. Louis, MO) was subcutaneously administered to mice on an escalating dose starting from 10 mg/kg up to 40 mg/kg twice per day for 4 days (Figure 1A) in 50–100 μl volumes of 0.9% NaCl as previously described [18, 19, 22].

### Suberoylanilide hydroxamic acid administration

Suberoylanilide hydroxamic acid (SAHA) was purchased from Cayman Biochemical (Ann Arbor, Michigan), which was dissolved in DMSO and diluted with saline (Final DMSO concentration 70%). Animals received SAHA 50 mg/kg or vehicle, daily via intra-peritoneal (i.p.) injection (100uL volume) concurrently with/out morning morphine treatment during the chronic dosing paradigm.

### ANA-12 and nor-BNI Administration

ANA-12, a BDNF receptor (tropomyosin related kinase B, TrkB) antagonist and nor-BNI (Norbinaltorphimine), a selective KOR (κ-opioid receptor) antagonist were purchased from Sigma Chemical (St. Louis, MO). ANA-12 and nor-BNI were dissolved in physiological saline. Animals received either drug (1 mg/kg for acute on days 5 and 12, or 0.5 mg/kg once daily for concomitant treatment with morphine) or vehicle, daily via i.p. injection 1 hour before testing (100uL volume). The selected drug doses and timing of administration were in accordance with prior publications [50–52].

### Dynorphin A and BDNF Administration

Intrathecal BDNF 1 ng/5uL, Dynorphin A 1 nmol (5mcl) or saline (5uL) were carried out in separate groups of mice 7 days after cessation of morphine/saline treatment. BDNF and Dynorphin A were obtained from Sigma Chemical (St. Louis, MO) and dissolved in saline. The animals were gently restrained prior to administration.

### Behavioral measurement

Nociceptive testing was done under treatment-blind conditions. Mechanical allodynia was assessed using nylon von Frey filaments according to the “up-down” algorithm described by Chaplan et al. [53] as previously described [19, 22, 48]. For these measurements, mice were placed on mesh platforms within transparent plastic cylinders. After 15 minutes of acclimation, nylon fibers of sequentially increasing stiffness were applied to the plantar surface of the hind paw which was left in place 5 sec. Withdrawal of the hind paw from the fiber was scored as a response. If no response was observed, the next stiffer fiber was applied to the same paw; if a response was observed a less stiff fiber was applied. Testing continued until 4 fibers had been applied after the first withdrawal response allowing the estimation of the mechanical withdrawal threshold. This data fitting algorithm allowed the use of parametric statistics for analysis [54].

### Chromatin Immune Precipitation (ChIP) Assay

Mice were sacrificed using carbon dioxide asphyxiation and spinal cord lumbar segments (L3-S1) were quickly dissected in pre-chilled surface. Tissue was flash frozen in liquid nitrogen and stored at -80°C until use. The assay was performed following the protocol adopted and refined previously by our lab [18]. Briefly, small pieces of minced tissue were cross-linked using 1% formaldehyde and sonicated on ice. The sonicated chromatin was clarified by centrifugation, aliquoted, and snap-frozen in liquid nitrogen. To perform ChIP, chromatin (150 μl) was diluted 10-fold and purified with specific antibody against aceH3K9 (Upstate Biotechnology, Waltham, MA) or IgG as negative control. Sonicated chromatin 1% was used for input control. The DNA that was released from the bound chromatin after cross-linking reversal and proteinase K treatment was precipitated and diluted in low-TE buffer (1 mM Tris, 0.1 mM EDTA). Quantitative PCR of target gene promoter enrichment in ChIP samples was done by ABI prism 7900HT system using SYBR Green. Five microliters of input ChIP or IgG sample were used in each reaction in duplicate for three biological samples in each condition. Fold enrichment was calculated as a ratio of the ChIP to mock IgG. An in-plate standard curve determined amplification efficiency (AE), and the 100 fold dilution factor for the input was included. Samples were then normalized to the saline condition.

### Quantification of mRNA

Spinal cord collection method was done as noted above and qPCR carried out as previously described [18]. Briefly, total RNA was extracted and RNA concentration was determined spectrophotometric analysis and complementary DNA was synthesized from total RNA using random hexamer priming and a first strand synthesis system (Invitrogen, Carlsbad, CA). Expression of genes of interest was determined by quantitative real-time PCR using ABI prism 7900HT system (Applied Biosystems). β-actin was used as an internal control and the expression level of specific genes was analyzed with ΔΔCt method.

### Immunohistochemistry

The technique of immunohistochemical analysis was described previously [55]. Briefly, the spinal cord was fixed in 4% paraformaldehyde for 24 h. Blocking of the sections took place at 4°C for 1 h in phosphate buffered saline containing 10% normal donkey serum, followed by exposure to the primary polyclonal antibodies against rabbit anti-BDNF antibody (1:100, Santa Cruz, Dallas, TX ), mouse anti-acetylated histone H3K9 antibody (1:1000, GeneTex, Irvine, CA), mouse anti-NeuN (1:500, Millipore) and mouse anti-GFAP (1:500, Millipore) overnight at 4°C. Sections were then rinsed and incubated with fluorescein-conjugated secondary antibodies against the primary antibodies (1:500, Jackson ImmunoResearch Laboratories, West Grove, PA) for 1 h. Double- labeling immunofluorescence was performed with donkey anti-mouse IgG conjugated with cyanine dye 3, or donkey anti-rabbit IgG conjugated with fluorescein isothiocyanate secondary antibodies. Confocal laser-scanning microscopy was carried out using a Zeiss LSM/510 META microscope (Thornwood, NY). Sections from control and experimental animals were processed in parallel. Control experiments omitting either primary or secondary antibody revealed no significant staining. The numbers of BDNF positive cells, NeuN (neurons) or GFAP (astrocytes) positive cells plus BDNF positive neurons/astrocytes were counted by a blinded experimenter in 10–15 randomly (2 slices per mouse) selected high-power fields (HPF, 400X) of spinal cord dorsal horn per animal.

### Enzyme immunoassay for BDNF and dynorphin levels in spinal cord

Mice were first euthanized by carbon dioxide asphyxiation and spinal cord tissue was harvested by extrusion. Lumbar spinal cord segments were dissected on a chilled surface. Dissected tissue was then quick-frozen in liquid nitrogen and stored at -80°C until required for analysis. Mice lumbar spinal cord were homogenized in ice cold 0.9% NaCl containing a cocktail of protease inhibitors (Roche Applied Science), centrifuged at 12,000G for 10 min at 4°C and then supernatant fractions were frozen at -80°C. An aliquot was subjected to protein assay (Bio-Rad) to normalize protein levels. BNNF concentrations were measured in duplicate by using mouse BDNF ELISA kit (GenWay Biotech), and the dynorphin levels were assayed in duplicate by using Dynorphin EIA kit (Phoenix Pharmaceuticals) according to the manufacturer’s instructions.

### Statistical analysis

All data are expressed as the means ± standard error of the mean (SEM) unless otherwise noted. The data for qPCR and ChIP experiments were analyzed by one-way analysis of variance (ANOVA) followed by Tukey’s post-hoc test for multiple comparisons. Data from the enzyme immunoassay were analyzed by two-way ANOVA followed by Tukey’s post-hoc test for multiple comparisons for each timepoint. The data for mechanical sensitivity were analyzed by one-way ANOVA followed by Sidak post-hoc test for multiple comparisons for each timepoint. Comparisons between 2 groups for immune-staining quantifications involved unpaired t-testing with two-tail p values. P < 0.05 was considered significant.
